# Signaling in time and space

**DOI:** 10.1186/1471-2164-15-S2-O18

**Published:** 2014-04-02

**Authors:** Norbert Perrimon

**Affiliations:** 1 Department of Genetics, Harvard Medical School, USA

## 

Organisms sense and interpret numerous internal and external signals by activating various signal transduction pathways. Past studies have illustrated that not only the presence or absence of one signal matters, but also that the frequency, amplitude, and duration of the signal provide crucial information to control many biological processes such as DNA repair, cell cycle, cell proliferation and differentiation. For example, oscillation of p35 activity is associated with cell-cycle arrest and recovery, whereas a single long-lasting activation of p35 leads to apoptosis. As a result, it is critical to have methods for detection of signal activity in vivo and in real time. Although the application of genetically encoded fluorescent proteins (FPs) as live signal reporters has revolutionized the field, most of the FP-based transcriptional reporters suffer from slow maturation (>1 hr) and extremely long half-life (>24 hr). This limitation greatly reduces the ability of these reporters to reveal the real-time dynamics of signals. To address this issue, new FP variants such as superfolder GFP (sfGFP) with a faster maturation time and high brightness have been engineered. In addition, the half-life of FPs can be shortened to about 2 hr by tagging them with a PEST domain for protein degradation. However, although short-lived FPs can report changes more faithfully, they have a major drawback in that they show a dramatic decrease in the fluorescent signal since much less FP is left for detection. I will discuss our recent efforts at developing new FP with improved temporal resolution.

In addition, our laboratory is exploring methods to interrogate the organization of protein complexes in polarized cells. Polarization of the epithelial cell defines its functions, including selective absorption, secretion, and transport. Along the apical to basal axis, at least five distinct membrane domains can be defined. Numerous methods have been developed to determine the protein composition of polarized plasma membrane domains in epithelia. However, results to date have been inadequate, and have yielded relatively few insights into the biogenesis or functional differentiation of these domains. I will discuss our recent efforts at implementing in vivo methods of live cell proteomics using engineered peroxidase.

